# Almond Allergy: An Overview on Prevalence, Thresholds, Regulations and Allergen Detection

**DOI:** 10.3390/nu10111706

**Published:** 2018-11-08

**Authors:** Giuseppina Mandalari, Alan R. Mackie

**Affiliations:** 1 Department of Chemical, Biological, Pharmaceutical and Environmental Science, University of Messina, Viale SS, Annunziata, 98168 Messina, Italy; 2 School of Food Science and Nutrition, University of Leeds, Woodhouse Lane, Leeds LS2 9JT, UK; A.R.Mackie@leeds.ac.uk

**Keywords:** almonds, allergy, thresholds, prevalence, detection, regulations

## Abstract

Food allergy has been on the increase for many years. The prevalence of allergy to different foods varies widely depending on type of food, frequency of consumption and geographic location. Data from the literature suggests that the prevalence of tree nut allergy is of the order of 1% in the general population. Almond is one such tree nut that is frequently eaten in many parts of the world and represents a potential allergenic hazard. Given the need to label products that contain allergens, a number of different methods of direct and indirect detection have been developed. However, in the absence of population-based threshold data, and given that almond allergy is rare, the sensitivity of the required detection is unknown and thus aims as low as possible. Typically, this is less than 1 ppm, which matches the thresholds that have been shown for other allergens. This review highlights the lack of quantitative data on prevalence and thresholds for almonds, which is limiting progress in consumer protection.

## 1. Introduction

Food allergy is defined as “an adverse health effect arising from a specific immune response that occurs reproducibly on exposure to a given food,” by the 2010 National Institute of Allergy and Infectious Diseases, National Institutes of Health (NIAID/NIH)-supported Guidelines for the Diagnosis and Management of Food Allergy [1,2,3]. The “Exposure” to food allergens can be by ingestion, skin contact, or via airborne particles. Tree nuts can give severe reactions, characterized by multi-systemic and respiratory symptoms. 

Since food allergy is considered a public health problem, an accurate assessment of its true prevalence is needed. Sicherer and Sampson [4] have highlighted the apparent increase in food allergy with documented rates as high as 10%. This increase seems to be related to a number of genetic, epigenetic and environmental risk factors. Peanut, tree nuts, fish, shellfish, egg, milk, wheat, soy, and seeds currently represent the major allergenic foods, mainly affecting industrialized/westernized countries, and more frequent in children than the adult population [2,3]. A number of hypotheses have been proposed in relation to the increased prevalence of food allergy, including the following risk factors: atopic dermatitis, increased hygiene/cleanliness with associated reduction to pathogen-exposure, microbiome changes, allergens avoidance in early life as well as dual allergen exposure, nutritional factors, such as vitamin D insufficiency, reduced antioxidants consumption and obesity [3,5,6,7].The aim of this review is to highlight the lack of reliable data on almond allergy, partially due to the fact that almond allergy is rare. We describe the data on the prevalence of tree nut allergy with particular focus on almond allergy, highlighting methods of detection of almond allergens, available regulations and limitations of available threshold data.

## 2. Prevalence

The first thing to note is that accurate data on prevalence is very limited and this is even more the case with individual tree nut species. Tree nut allergy rates vary by geographical region and depend on the type of nuts consumed within that region, with ethnic differences reported in the prevalence of cashew nut, pistachio nut and almond allergy in the UK [8]. The true prevalence of tree nut allergies is unknown, but in the US the rate is estimated to be 0.2% and 0.5% for children and adults, respectively [9]. A recent review by McWilliam et al. [10] estimated tree nut to cause around 0–0.19% of food allergies across the world. Of the 36 studies identified in that review, most were in children (*n* = 24) and from Europe (*n* = 18), UK (*n* = 8) or USA (*n* = 5). 

Prevalence of self-reported tree nut allergy in Canada was 1.2% in the general population and 1.1% in adults [11]. Lower rates were observed in Australian adults [12]. 

Due to cross-reactivity, data for individual nuts is more limited, though Wan and Chiu [13] reported that 2.2% of Taiwanese children (age 6 to 8) were allergic to pistachios. In the US, almonds are the third most common tree nut to cause allergy after walnuts and cashews [14,15]. In Europe, the prevalence of self-reported allergy to any nut was estimated to be 1.7% in the general population (UK) [16], ranging from 0.1% in children (Turkey) [17], to 1.3% (The Netherlands) [18] and 6.9% (Spain) [19] in children. Hazelnut allergy was the most common tree nut allergy [20]. Prevalence data for almond allergy are almost exclusively available only for children.

It seems clear that tree nut allergy rates vary by geographical region and depend on the mix of nuts commonly consumed [21]. Geographical comparisons are difficult to make since country-based studies differ in the type of nut, age group and method of diagnosis [22]. 

It is believed that allergy to one type of nut is a risk factor for developing allergy to other types of nuts. Multiple tree nut allergies were found in 19% of 2 years old children and 86% of children at 5 to 14 years [23]. Similarly, reaction to multiple nuts was recorded in 2% of 2 year old children and 47% of 14 year olds [24]. 

A recent investigation showed a cherry seed-derived spice, *mahleb*, is recognized by anti-almond antibodies including almond-allergic patient IgE [25]. The cross-reactivity between almond and *mahleb*, which is prepared from the kernels of a species of cherry, should be of particular concern to almond-allergic patients and attending medical personnel. Uotila et al. [26] reported that birch-sensitized individuals are frequently co-sensitized to the Bet v 1 homologues (PR10 proteins) in hazelnut, almond and peanut, although the prevalence decreases from early childhood to adolescence. Cashew and pistachio, and pecan and walnut were the most widely seen cross-reactions. Tree nut challenges are also frequently passed in patients with tree nut sensitization, and nearly all patients with peanut allergy and tree nut co-sensitization passed the challenge, thus calling into question the clinical relevance of cross-reactivity [27].

A recent study reported that household consumption of almond and peanut was related to development of sensitization to peanuts [28].

According to the European Academy of Allergy and Clinical Immunology (EAACI) Molecular Allergology User’s Guide [29], five types of clinical patterns and relevance have been described amongst individuals sensitized or allergic to tree nuts and seeds: (1). Primary allergy and sensitization to one tree nut or seed allergen (IgE level low, generally young patients, ie cashew nut allergy); (2). Co-sensitization to at least 2 primary tree nuts and/or seed allergens (relatively high IgE levels); (3). Primary sensitization and allergy to at least one tree nut or seed associated to cross-reactive IgE to another botanically related tree nut or seed (i.e., patients sensitized to both cashew and pistachio or to walnut and pecan with equal IgE levels); (4). Primary sensitization and allergy to at least one tree nut or seed and cross-reactive IgE to another botanically not closely related tree nut or seed (i.e., patient with allergy to a few nuts with high IgE and with IgE to other nuts 10 times less or more); (5). Primary sensitization to pollen and cross-reactive IgE birch-pollen related food allergy. 

The studies outlined above highlight the diversity of prevalence data and the limitations of sensitization as a measure of allergy. Both are factors that make risk factors for almond allergy very difficult to calculate. 

## 3. Almond Allergens

Almond proteins, including the major storage protein amandin, have been identified as allergens. Eight native almond (*Prunus dulcis*) allergens have been characterized according to their biochemical function as summarized in Table 1 [30,31,32,33]. However, only four, Pru du 3, Pru du 4, Pru du 5, and Pru du 6 (amandin) are recognized and included in the WHO−IUIS list of allergens [34]. 

Pru du 6, an 11S globulin also known as amandin, was one of the first allergens to be studied in almonds and has been defined both as a major storage protein and as a major allergen, accounting for 65% of total almond protein content. Pru du 6 has been related to severe reactions to almond upon ingestion [35]. The Pru du 6 isoforms have been sequenced, cloned and screened for IgE binding in almond-allergic patient sera [36]. The results of these isoform studies showed that of the two isoforms, Pru du 6.01 is more widely recognized than Pru du 6.02 in almond allergic subjects and denaturing of Pru du 6 isoforms had little effect on IgE-binding intensity in some sensitive subjects, suggesting an important role for sequential epitopes on the sub-units of Pru du 6 [36]. Other conformational epitope mapping studies of Pru du 6 have been performed with a murine monoclonal antibody (mAb) 4C10 [37]. Polypeptides from Pru du 6 are highly resistant to different heat treatments during food processing, and the contamination of food with this allergen can lead to a significant risk for sensitized patients [38]. Pru du 6 was found to be sensitive to pepsin during simulated gastric digestion using an in vitro model of the gastrointestinal tract [39]. However, incorporation of almond flour into a food matrix, such as chocolate mousse or Victoria sponge cake, decreased the rate of Pru du 6 degradation by pepsin. Immunoreactivity of almond polypeptides detected by dot blots and sandwich enzyme-linked immunosorbent assay (ELISA) retained better reactivity [39]. In a study by Holden et al. [40], it was suggested that Pru du 6 could possibly cross-react with α-conglutin from lupine, since this protein is another 11S globulin. The seed storage proteins 2S albumin and γ-conglutin were characterized as IgE-binding proteins [41]. Sera from almond allergic patients, reactive to skin prick tests and showing positive-response to almonds in oral challenge tests, allowed the isolation of the two almond allergens (2S albumin and γ-conglutin). However, the IgE binding and the serological reactivity to these proteins did not imply any clinical symptoms, and further studies are needed. As a result, neither of these have been incorporated into the standard clinical nomenclature. 

Pru du 5, also known as 60S Acidic Ribosomal Protein P2, has been described as an almond allergen [42]. The immunoreactivity with ELISA using pooled and individual serum from almond allergic individuals showed that the expressed Pru du 5 proteins possessed the ability to bind IgE antibodies. However, further investigation is needed in order to classify Pru du 5 as a major allergen.

### 3.1 Detection of Almond Allergens

Several methods have been developed for almond allergen detection, mainly based on immunochemical, DNA-based techniques, and mass spectrometry (MSs) [30].

#### 3.1.1. Immunochemical Methods 

Immunochemical methods have been used to detect allergens in food by precise binding between epitopes present on the target protein and an immunoglobulin. These methods, primarily ELISAs, have become the standard for qualitative and quantitative detection of allergen in food products [43,44]. ELISA detects protein(s) and is sufficiently sensitive (detection limits of parts per million), thus providing rapid assessments [43]. A number of immunochemical methods have been used for almond allergen detection, including ELISA, lateral flow devices (LFD) and immunoblotting.

##### ELISA Methods 

These tests provide rapid, versatile and reliable testing of almond allergens with a limit of detection (LOD) down to 0.1 mg/kg (ppm) of almond protein in food samples within 30−35 min [45,46,47]. This is because Pru du 6 is water-soluble and has high thermal stability in foods [30,35,47,48]. ELISA successfully detected almond Pru du 6 residues in a wide variety of test foods including dark, milk and white chocolate, peanut butter, spices, cereal, cheese, ice cream, salt, cauliflower, powdered mango and others [49]. The Pru du 6 recovery range was 116–198 μg/100 μg (dairy), 110–292 μg/100 μg (tree nuts), 43–304 μg/100 μg (legumes), 106–183 μg/100 μg (most cereals—with the exception of barley, whole-wheat flour, wild rice and raisin bran whole mix). Pru du 6 recovery from spices was typically low (2–85 μg/100 μg) with a few exceptions where higher recoveries were observed (121–334 μg/100 μg). Salt (black and white), tea, confectionery (sugar, cocoa, dark chocolate), and fruits (1–83 μg/100 μg) generally resulted in lower recoveries. These data confirm that food matrix and extraction conditions affect immunoassays [50], in this case for Pru du 6 detection and quantification. Research by the United States Department of Agriculture (USDA) showed that pulsed UV-light on liquid peanut butter can reduce ELISA peanut allergen binding by about 7-fold [51]. This pulsed-light treatment may have application for reducing the allergenicity of specialty almond products like almond milk. 

Indirect sandwich ELISA has been used to detect almond protein (8 μg) spectrophotometrically at 405 nm: Pru du 6 was identified in almond flour dispersed in water or included into a food matrix such as chocolate mousse and Victoria sponge [39]. A murine-monoclonal antibody (mAb)-based ELISA for almond detection has recently been developed [52]. Pru du 6 presence in 108 almond genotypes/hybrids and 80 almond marketing varieties grown in different locations was determined using murine- mAb 4C10-based sandwich ELISA. The results indicated that Pru du 6 was present in all of the samples tested. The ELISA immunoreactivity variations were up to 8-fold among genotypes/hybrids and 2.5 fold amongst the almond marketing varieties. Another study has reported the development of a sandwich ELISA using anti-almond soluble protein rabbit polyclonal antibodies as capture antibodies and murine-mAb as detection antibodies [53]. The assay was specific and sensitive (3–200 ng almond protein/mL) for almond detection. The standardized assay is accurate (<15% CV) and reproducible (intra- and inter assay variability <15% CV). The assay did not register any cross-reactivity with the food matrices tested, suggesting the assay to be almond Pru du 6 specific. The assay could detect the presence of declared almond in the tested matched commercial samples. Further, the assay reliably detected the presence of almonds in the laboratory-prepared food samples spiked with almond flour. A recent publication by Su et al. [54] reported the use of a murine-mAb linked with ELISA to assess Pru du 6 immunoreactivity in processed and long-termed stored almonds. The results demonstrated that Pru du 6 immunoreactivity is stable in processed almond seeds and could be detected in whole raw and processed (blanched, sliced, dry-roasted, flour and defatted flour) almonds for several years.

##### Lateral Flow Devices or Dipstick Assays 

These are based on the same principle as ELISA but are simpler and faster (about 10 min) and used by industry for rapid allergen screening [30]. The results are primarily qualitative or semi-quantitative so the drawbacks with this method are the potential for false negatives and lack of quantitative data. However, these tests provide quick on-site detection of almond allergens within minutes. For example, detection down to 1 ppm in 10 min. 

##### Immunoblotting

This is also based on ELISA principles and is very reliable but not for routine analysis [30]. Immunoblotting constitutes a confirmatory test for the presence of almond allergens in food. Immunoblotting can detect almond protein in chocolate with a LOD of 5 ppm [55]. A novel immunoassay test system was developed to detect modified allergen residues present in almond, cashew, coconut, hazelnut and soy-based non-dairy beverages [56]. The tests showed robust detection capabilities, a sensitivity of 1 ppm and selectivity values of 3–5 ppm. 

#### 3.1.2. DNA-Based Methods

These methods do not directly target protein allergens but rather amplify the gene fragment encoding for the allergen protein by means of polymerase chain reaction (PCR), either as qualitative endpoint PCR or as quantitative real-time PCR assays. DNA-based methods take advantage of the greater thermal stability of DNA molecules compared to proteins. This method enables the detection of low quantities of almond DNA (5 pg), with LOD ranging from 1 to 100 mg/kg of almond in spiked biscuits [57]. Methods combining both PCR and ELISA have been developed for the detection of food allergens to fit standards for potential labeling requirements [58]. However, since PCR tests do not detect proteins from their source, their use in food allergy risk assessment is confined [59].

#### 3.1.3. Mass Spectrometry (MS)-Based Methods

The MS methods provide specific information on the identification of allergenic proteins in almonds [60]. These methods rely on the primary sequence of proteins and peptides with data on molecular mass and protein identification algorithms. The LOD for specific almond allergens is 3 ppm in bread [30]. Advanced MS techniques have recently been used for the isolation and characterization of a potentially allergenic Pru du 3 (lipid transfer protein) in almond: the full sequence was identified with LC-ESI-Orbitrap-MS and consisted of 92 amino acids, with a predicted molecular weight of 9579.0 [61]. 

The use of MS methods for the qualitative and quantitative detection of allergenic food proteins has recently been further explored [62]. Like ELISA, MS methods are able to identify the allergenic proteins, thus providing a direct evaluation for risk assessment. MS methods can also detect multiple food allergens simultaneously [63], with a sensitivity similar to ELISA methods. However, MS is not as widely available as ELISA, but will most likely be used more in the future.

#### 3.1.4. Allergen Microarrays

Allergen microarrays contain a large variety of allergen molecules and thus allow the simultaneous detection of allergic patients’ antibody reactivity profiles towards each of the allergen molecules with only minute amounts of serum. Advances in the allergen-microarray technology for diagnosis and monitoring of allergy have recently been discussed through the use of a MeDALL allergen-chip [64]. The MeDALL allergen-chip has been developed for the specific and sensitive monitoring of IgE and IgG reactivity profiles towards more than 170 allergen molecules in sera collected in European birth cohorts. MeDALL is a European research program in which allergen microarray technology is used for the monitoring of the development of allergic disease in childhood, to draw a geographic map of the recognition of clinically relevant allergens in different populations and to establish reactivity profiles, which are associated with and predict certain diseases. 

#### 3.1.5. Adenosine Tri-Phosphate and Total Protein Methods

Other methods, such as the Adenosine Tri-Phosphate (ATP) test and total protein tests, are used by the food industry [43]. These methods are useful tools for monitoring the cleaning process, but do not provide any quantitative detection for risk assessment.

## 4. Threshold Dose Distribution and Precautionary Allergen Labelling 

Thresholds are minimum allergen concentrations present in a food which trigger a reaction in a sensitized person. It is very challenging to establish the amount of an allergenic protein that poses a health risk. Levels vary according to the allergen and an individual’s sensitivity [65]. Significant amounts of such data are needed in order to provide population-based threshold data. Since 2005, several clinical investigations have been performed with low dose challenges in order to provide data for modeling purposes [66,67]. The EuroPrevall project, the largest multicenter European study on food allergy, aimed to develop effective management strategies in food allergy through a multidisciplinary integrated approach [68]. Threshold dose distributions for 5 major allergenic foods in the European populations were defined [69]. Low-dose, double-blind, placebo-controlled food challenges (DBPCFCs) were undertaken with commercially available food ingredients (peanut, hazelnut, celery, fish and shrimp) blinded into common matrices. Of the 5 foods used for challenge, 4 (peanut, hazelnut, celery and fish) produced similar dose distributions, with estimated doses eliciting reactions in 10% of the allergic population ranging from 1.6 to 10.1 mg of protein for hazelnut, peanut and celery and 27.3 mg of protein for fish. Standardized DBPCFCs were also used to confirm thresholds data for milk, egg, fish, shrimp, peanut, hazelnut, celeriac, apple and peach [68]. Currently, no threshold doses are available for almonds.

Food matrix and other extrinsic factors could affect threshold doses. It is becoming clear that, as the major mucosal surface comes into contact with the food we eat, the mucosal barrier of the gastrointestinal tract plays an important role in both the development of food allergy (sensitization) and manifestation of an allergic reaction (elicitation). Therefore, the effects of digestion may help explain and predict effects of the food matrix on the delivery of allergen to the mucosal surface, including how the food matrix may modulate allergic reactions [70,71]. 

Food processing also affects allergenic potential [72,73]. The types of processing implicated in influencing allergenic properties are the following: heating (thermal processing), fermentation including endogenous enzymatic hydrolysis, enzymatic and acid hydrolysis, physical treatment (such as high pressure processing or extrusion), the use of preservatives, changes in pH, or combination of any two or more of these [23,74]. 

In almonds, blanching and roasting did not have any effect on the allergenicity of Pru du 6, confirming the heat stability of this protein. However, using immunoblotting with sera from almond allergic patients, blanching and roasting reduced the IgE-binding of a 15–17 KDa band, which may be Pru du 1 [38,75]. De Leon et al. [76] found no difference in IgE-binding (ELISA) between roasted (180 °C) and unroasted almonds in the serum from one almond allergic patient. The difference might be due to the small size of sample tested in the studies.

Su et al. [77] demonstrated no changes in the immunoreactivity of Pru du 6 after γ-irradiation and γ-irradiation plus thermal treatment. However, Dhakal et al. [78] reported a decreased immunoreactivity of Pru du 6 after high-pressure treatment in almond milk, whereas no significant change was detected after thermal treatment. The effects of dry and moist heat, autoclave sterilization and high-pressure treatment on the biochemical and immunological properties were also evaluated [79]. Results showed that almond proteins in food products were stable during dry-heat treatment at temperatures below 250 °C, whereas the combined effect of heat, pressure and presence of water resulted in a significant change of solubility and immunoreactivity of almond proteins.

A draft scientific opinion on the evaluation of allergenic foods and food ingredients for labelling purposes published recently by the European Food Standard Authority (EFSA) made the following statement: “*Most studies available report on the IgE-binding capacity of processed foods rather than on their allergenicity, whereas systematic investigations on the effects of food processing on allergenicity are scarce*” [23].

All stakeholders agree that precautionary allergen labelling must reflect actual risk and should indicate the likely unintended presence of an allergen in a consumed portion of a food product that is at or above a reference dose [80]. There is a general duty of care on the food industry and the obligations in European Union (EU) legislation to reduce and manage the presence of allergens. While there is an EU regulation for allergen present as an ingredient, this is not the case for the unintended presence of allergens [81]. There is an urgent requirement for effective communication between healthcare professionals, patient organizations, food industry and regulators in order to develop a better approach to protect consumers with food allergies [82].

## 5. Conclusions

In this review, we have looked at the evidence for the prevalence to almond allergy, which shows that almond allergy is rare. Although almond has 4 primary allergens, of which Pru du 6 is the most studied, there is little evidence for the efficacy of component-resolved diagnosis. In terms of detection, the immunoassay-based methods are still the most widely used and seemingly the most reliable in food-based products, although the potential of MS methods might lead to promising sensitivities once more almond proteins have been sequenced and the available database updated accordingly. Our primary conclusion is that population-based threshold data is needed in order to provide effective risk assessment and consumer advice. Thus, more studies need to be undertaken with the relevant allergic groups so that we can move beyond the current precautionary labelling.

## Figures and Tables

**Table 1:** Potential Almond Allergens [30,36].

Name/Protein Family	Molecular Weight/Structural Details	Biological Function	Food Processing Effects	Clinical Relevance
Pru du 1 (PR-10 Protein)	17 kDa (160 amino acids); various isoforms with different IgE binding capability	Protects against pathogens and adaption to a stressful environment	Wet heat processing changes eptitope conformation to reduce IgE reactivity	Mild immune reaction; severe allergic reactivity with birch pollen allergy
Pru du 2 (PR-5 Protein/thaumatin-like protein)	23–27 kDa (246 amino acids)	Response to pathogen infection, osmotic stressor fungal proteins	Very resistant to protease, pH, or heat treatment	Recognized as potential potent allergen, but the clinical evidence has not been studied
Pru du 2S albumin (prolamin super family)	12 kDa (28 amino acids)	Nut storage protein for seed development	Stable to heat treatment	Specific allergic symptoms not yet defined, more studies needed
Pru du 3 (prolamin super family)	9 kDa (116–123 amino acids)	Lipid transfer protein and defensive system against bacteria and fungi	Very resistant to pH, thermal and enzyme treatments	Systemic and life-threatening symptoms; cross reactivity among *Rosaceae* fruit
Pru du 4 (profilin-specific IgE)	14 kDa (131 amino acids)	Actin-binding protein for cellular function	Labile protein during heat processing	Symptoms are mild and limited to oral cavity
Pru du 4 (profilin-specific IgE)	14 kDa (131 amino acids)	Actin-binding protein for cellular function	Labile protein during heat processing	Symptoms are mild and limited to oral cavity
Pru du 5 (r60sRP autoimmune reactions to human P2)	10 kDa (113 amino acids)	Involved in protein synthesis	Unknown	Specific allergic symptoms not yet defined, more studies needed
Pru du 6 (amandin; almond major protein (AMP)) The most widely studied almond allergen regarding molecular structure and biochemistry.	360 kDa (1055 amino acids); 11S globulin hexameric amandine polypeptides subunits of 40–42 kDa acid α chain and a small β chain linked by a disulfide bond.	Major storage protein (about 65% of almond protein)	Thermally stable to dry heat such as roasting but it can be denatured by boiling.	Reported to induce severe IgE allergic reactions
Pru du (γ-conglutin)	45 kDa for each subunit (25 amino acids)	7S vicillin storage protein	Unknown	Specific allergic symptoms not yet defined, more studies needed
